# Locais de Reconexão na Técnica de Reablação após Isolamento das Veias Pulmonares com Criobalão em Pacientes com Fibrilação Atrial Paroxística

**DOI:** 10.36660/abc.20190503

**Published:** 2021-07-15

**Authors:** Rogelio Robledo Nolasco, Gerardo De Leon-Larios, David Eduardo Bazzini-Carranza, Elias Zavaleta, Omar Calixto-Vargas

**Affiliations:** 1 Centro Medico Nacional 20 de NoviembreCiudad de MéxicoMéxicoCentro Medico Nacional 20 de Noviembre - Hemodinamia y Electrofisiologua, Ciudad de México – México

**Keywords:** Arritmias Cardíacas, Fibrilação Atrial, Técnicas de Ablação, Veias Pulmonares, Ondas de Rádio, Eletrocardiografia/métodos, Eletrocardiologia Ambulatorial, Taquicardia Atrial Ectópica, Holter

## Abstract

**Fundamento:**

Na fibrilação atrial paroxística (FAP), o isolamento das veias pulmonares com criobalão (IVP-CB) tem eficácia semelhante à da ablação por radiofrequência (IVP-RF). Em procedimentos de reablação após IVP-RF, a reconexão das VPs é alta, ao passo que em pacientes com reablação após IVP-CB, as informações são escassas.

**Objetivo:**

Determinar os locais de reconexão das VPs em pacientes que foram submetidos à reablação após IVP-CB inicial.

**Métodos:**

Pacientes que foram submetidos a um procedimento de reablação de fibrilação atrial, após um IVP-CB inicial para FAP foram incluídos. O mapeamento eletroanatômico do AE foi utilizado. Um local de reconexão foi definido com a presença de uma voltagem de 0,3mV ou maior nas VPs e condução unidirecional ou bidirecional nas VPs durante o ritmo sinusal. Os locais de reconexão foram identificados por meio de corte paraesternal longitudinal e posteriormente ablacionados com radiofrequência.

**Resultados:**

Dos 165 pacientes submetidos ao IVP inicial, 27 necessitaram reablações, dos quais 18 (66,6%) eram do sexo masculino, com média de idade de 55+12,3 anos. O tempo de recorrência foi de 8,9+6,4 meses. A reconexão das VPs foi encontrada em 21 (77,8%) pacientes. Houve um total de 132 lacunas de condução, seis por paciente, 3,6 por VP. Um número significativo de lacunas ocorreu na região ântero-superior da VP superior esquerda (VPSE) e nas regiões septal e inferior da VP superior direita (VPSD).

**Conclusões:**

As VPs superiores apresentaram os locais de maior reconexão, principalmente na região anterior da VPSE e na região septal da VPSD. A razão por trás disso pode ser devido à maior espessura da parede atrial e à dificuldade em alcançar o contato de criobalão adequado.

## Introdução

O isolamento das veias pulmonares (IVP) é o fundamento da terapia de ablação na fibrilação atrial (FA) paroxística e persistente. As técnicas mais comuns para obter IVP são a ablação com criobalão (CB) e a ablação por radiofrequência ponto a ponto, ambas com resultados semelhantes.^1,2^ A recorrência de FA após o procedimento inicial de IVP considera-se como sendo mediada pela reconexão da veia pulmonar (VP), visto que 80% dos pacientes submetidos à reablação apresentam recuperação da condução da VP em pelo menos um local.^3-5^ Um estudo para determinar a presença de reconexões das VPs em pacientes submetidos à reablação após IVP-CB inicial foi realizado. Além disso, descreveram-se os locais com maior probabilidade de recuperação da condução da VP.

## Métodos

Pacientes sintomáticos com FA que apresentaram recorrência após um IVP com crioablação inicial, e que foram submetidos a um procedimento de reablação, foram incluídos no estudo. Os pacientes que receberam terapia inicial de crioablação inicial foram aqueles com FA resistente a fármacos com fração de ejeção do ventrículo esquerdo (FEVE) preservada, um diâmetro ântero-posterior (AP) do átrio esquerdo de 55 mm ou menos e nenhuma evidência de trombos no apêndice atrial esquerdo (AE) em um ecocardiograma transesofágico.

O procedimento foi realizado sob sedação consciente, e as veias femorais foram utilizadas como acesso venoso. Um cateter decapolar (Webster® Decapolar Catheter Deflectable) foi posicionado no seio coronário e uma punção transseptal atrial guiada por EIC foi realizada utilizando uma bainha 8F (Preface Braided Guiding Sheath) e uma agulha de Brockenbrough (Agulha Transseptak BRK). Uma bainha orientável 12F (FlexCath®sheath Medtronic, Minneapolis, MN, EUA) e um cateter de mapeamento circular (Achieve Medtronic) foram então inseridos no AE. Um cateter de balão de crioablação de 28 mm (Arctic Front, Cryocath^TM^, Medtronic, CA, EUA ou Arctic Front Advance, Minneapolis, MN) foi utilizado para administrar a terapia de crioablação no ântero de cada veia pulmonar. A terapia de crioablação foi administrada por 180 a 300 segundos até que uma temperatura mínima de -40ºC fosse atingida e o IVP assegurado. A terapia foi considerada bem-sucedida se o bloqueio de entrada e saída da VP foram ambos alcançados. Durante a isolação da VP direita, o cateter quadripolar foi posicionado na veia cava superior para estimulação contínua do nervo frênico em ciclo de 1.800 ms e saída de 20 mA para evitar paralisia do hemidiafragma.

Durante o acompanhamento, foram prescritos eletrocardiograma e monitor de Holter de 24 horas (ou marca-passo) três meses após o procedimento e depois de seis meses adicionais. A medicação antiarrítmica foi suspensa após os primeiros três meses se nenhuma FA ou taquiarritmia atrial fosse identificada. A recorrência foi definida com a presença de FA ou outra taquicardia atrial, em tira de eletrocardiograma ou durante pelo menos 30 segundos em Holter após a primeira consulta de acompanhamento.

O procedimento de reablação foi realizado sob sedação consciente, por meio de acesso da veia femoral e apenas uma punção transseptal atrial. O anticoagulante não foi suspenso para a realização do procedimento de ablação. Sistema Carto 3® (Biosense Webster, Diamond Bar, CA, EUA) e um cateter multipolar (Pentaray Nav, BiosenseWebster) foram utilizados para construir mapas de tensão do AE e de cada VP, adotando <0,3mV como valor de corte para tecido cicatricial e >1,0mV para tecido normal. O isolamento da VP foi definido como a ausência de atividade elétrica dentro da VP durante o ritmo sinusal ou FA e/ou a presença de bloqueio de entrada e saída se o paciente estivesse em ritmo sinusal.

Os locais com lacunas de condução foram identificados por meio de corte paraesternal longitudinal, sendo considerados 12 locais diferentes. Uma vez localizados os locais de reconexão das VPs, eles foram ablados com radiofrequência utilizando um cateter de radiofrequência baseada em sensor de força de contato irrigado (ThermoCool SmartTouch, Biosense Webster). Além disso, eletrogramas fragmentados foram ativamente pesquisados e marcados, principalmente na parede posterior e teto do AE. Se o paciente estivesse em ritmo sinusal, a estimulação atrial a uma duração do ciclo de 170ms era adotado para induzir FA a fim de buscar eletrogramas fragmentados, que eram posteriormente ablacionados. A ablação da parede anterior e rebordo foi feita a 40 W, ao passo que na parede posterior foi a 25 W, com temperatura limite de 45ºC. A taxa de infusão de 17 a 30 mL/min de solução salina normal e uma força de pressão de 6-30g foram utilizadas.

Se o paciente estava em FA, a ablação foi considerada bem-sucedida se houvesse uma queda de impedância de 8-10 ohms, uma diminuição na amplitude ou eliminação de eletrogramas atriais. Se o paciente estava em ritmo sinusal, o sucesso da ablação foi determinado pela perda da captura de estimulação no local da ablação. Por fim, linhas de ablação foram feitas no teto e istmo mitral, e se *flutter* atrial típico fosse detectado, a ablação do istmo cavotricuspídeo também era realizada.

### Análise estatística

Este é um estudo observacional descritivo. A distribuição dos dados foi testada com o teste de normalidade Shapiro Wilk. As variáveis categóricas foram expressas em número total e porcentagens, ao passo que as variáveis com distribuição normal foram expressas como média e desvio padrão. O programa SPSS v.20 foi utilizado para análise de dados. Significância estatística foi considerada se p<0,05.

## Resultados

### População do estudo

De 2014 a 2018, 164 pacientes foram submetidos a IVP com a técnica de crioablação com balão, dos quais 27 apresentaram recorrência de FA e precisaram ser submetidos à reablação, após um acompanhamento de 10,7+7,2 meses. Desses pacientes, 18 (66,6%) eram homens com idade média de 55±12,3 anos, escore CHA2DS2-VASc médio de 1,9±1,6 e FEVE média de 60,8±17,2%. Características adicionais dos pacientes podem ser observadas na Tabela 1. Foi necessário consentimento informado e por escrito antes do procedimento de reablação de FA.

**Tabela 1:** – Características dos pacientes

Homens	18 (66,6)
Idade (anos)	55±12,3
Hipertensão	15 (55,5)
Diabetes mellitus	8 (29,6)
Insuficiência cardíaca	1 (3,7)
Histórico de infarto	1 (3,7)
Histórico de AVC (%)	3 (11,1)
Portador de marca-passo (%)	3 (11,1)
Duração da FA (meses)	13,2±13,5
CHA2DS-VASC	1,9±1,6
Quantidade de fármacos antiarrítmicos testados	1,2±0,6
FEVE	60,8±17,2
Diâmetro do AE (mm)	40,2±8,0
Tempo antes da recorrência (meses)	8,9±6,4

*Os dados são expressos em números (%) ou média ± desvio padrão. AVC: acidente vascular cerebral; FA: fibrilação atrial; AE: átrio esquerdo; FEVE: fração de ejeção do ventrículo esquerdo. A análise dos dados foi realizada com o programa SPSS v.20.*

### Técnica de reablação e reconexão de VPs

As características de procedimentos de reablação estão descritas na Tabela 2. O tempo para recorrência de FA foi de 8,8±8,2 meses. Dos 27 pacientes estudados, a recorrência de FA foi detectada em 17 (62,9%) deles, por monitoramento de Holter, sete (25,9%) por ECG e três (11,1%) por monitoramento do marca-passo. Além disso, 18 (66,6%) estavam em ritmo sinusal; os outros estavam em FA. Um local de reconexão foi definido com a presença de uma voltagem de 0,3mV ou maior na VP e condução unidirecional ou bidirecional na VP durante o ritmo sinusal.

**Tabela 2:** – Características do procedimento de reablação

Pacientes com reconexão de VP	21 (77,8)
Tempo antes da recorrência (meses)	10,5±6,5
Número de veias por paciente	1,6±0,4
Número de lacunas por paciente	6,0±0,5
Número de lacunas por VP	3,6±0,3
Lacunas da veia pulmonar superior esquerda	56 (42,4)
Lacunas da veia pulmonar inferior esquerda	12 (9,1)
Lacunas da veia pulmonar superior direita	35 (26,5)
Lacunas da veia pulmonar inferior direita	29 (21,9)
Ablação adicional realizada	
Eletrogramas de complexos atriais fracionados	4 (14,8)
Isolamento do istmo cavotricuspídeo	3 (11,1)
Tempo total do procedimento (minutos)	130±17
Tempo de fluoroscopia (min)	8,5±1,7

*Os dados são expressos em números (%) ou média ± desvio padrão. A análise dos dados foi realizada com o programa SPSS v.20. VP: veia pulmonar.*

Três acessos venais foram obtidos por veias femorais. Em todos os casos, foi realizada apenas uma punção transseptal guiada por ultrassom intracardíaco; primeiro, o cateter Pentaray foi introduzido para realizar um mapeamento de tensão e condução do AE e VP. 109 VPs foram identificadas nos 27 pacientes submetidos à reablação, dos quais 36 (33,0%) tinham pelo menos um local de reconexão. 22 pacientes (81,5%) apresentavam pelo menos uma VP com lacuna de condução, com média de 1,6±0,4 VP por paciente. Nove pacientes apresentaram um local de reconexão em uma VP (40,9%), 11 pacientes (50%) em duas VPs diferentes, um paciente (4,5%) em três VPs e um paciente (4,5%) em todas as quatro VPs.

### Localização das lacunas de condução em VP

**Figura 1:**
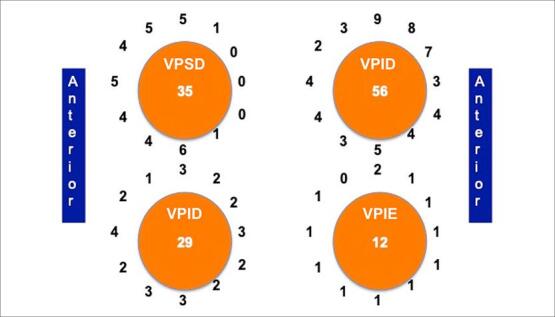
– Distribuição das lacunas de reconexão nas quatro VPs (o número no centro de cada círculo). A VPSE é a mais reconectada, 71% das lacunas ocorrem nas regiões ântero-superior e ântero-inferior. A VPSD teve mais reconexões nas regiões ântero-superior e septal. VPIE: veia pulmonar inferior esquerda; VPSE: veia pulmonar superior esquerda; VPID: veia pulmonar inferior direita; VPSD: veia pulmonar superior direita.

Um total de 132 lacunas de condução em IVP foi observado, com uma média de 6,0±0,5 lacunas de condução por paciente e 3,6±0,3 lacunas por VP; essas reconexões foram localizadas nos seguintes locais: 56 (42,4%) na VP superior esquerda, 35 (26,5%) na VP superior direita, 29 (21,9%) na VP inferior direita e 12 (9,1%) na VP inferior esquerda (Figura 1). O local com mais reconexões foi a junção da VPSE com o apêndice atrial esquerdo (rebordo endocárdico), seguido pela região posteroinferior da VPSE (71%); e, por último, a região posterior da VPSE (29%). A VPIE tinha menos lacunas de conexão, que foram distribuídas uniformemente ao redor da veia. A VPSD apresentou o maior número de reconexões fora das veias pulmonares direitas, principalmente nas regiões ântero-superior e inferior (94% do total). As lacunas da VPID foram distribuídas uniformemente ao redor da veia, favorecendo levemente as regiões inferiores.

### Localizações fragmentadas de eletrogramas e outras arritmias

Eletrogramas atriais fragmentados foram identificados em oito (29,6%) pacientes, principalmente na parede posterior. O *flutter *atrial típico foi encontrado em nove (33,3%) pacientes, submetidos à ablação do istmo cavo-tricuspídeo até a obtenção do bloqueio bidirecional.

A duração média dos procedimentos de reablação de RF foi de 130±17 minutos, com um tempo médio de fluoroscopia de 8,5±1,7 minutos. Ocorreram duas complicações relacionadas ao procedimento, um hematoma inguinal submetido a tratamento conservador e um derrame pericárdico que foi prontamente resolvido após a punção pericárdica.

## Discussão

O isolamento da veia pulmonar (IVP) por radiofrequência (RF) ponto a ponto demonstrou ser um tratamento eficaz para FA paroxística e, como tal, é atualmente recomendado nas diretrizes clínicas de FA.^6^ O fundamento da terapia de ablação de FA paroxística é o IVP, que tradicionalmente tem sido realizado com radiofrequência.^7-9^ Mais recentemente, a crioablação surgiu como alternativa viável, com resultados semelhantes.^1,2^ No estudo de Fogo e Gelo, ambas as abordagens foram igualmente eficazes, especialmente ao comparar o criobalão de segunda geração com o cateter de força de contato.^10^

Em pacientes submetidos a um procedimento de reablação após um IVP inicial, lacunas na conexão das VPs podem ser encontradas em mais de 95% das vezes. As veias pulmonares esquerdas parecem ter mais reconexões, especialmente nas áreas anterior e inferior.^4,11,12^ Katering et al.,^13^ publicaram uma série de casos de procedimentos de reablação de FA, documentando uma média de 2,9 reconexões das VPs em comparação com nossos achados, 1,6. Recentemente, outro estudo publicado mostrou um tempo de recorrência após IVP com crioablação e radiofrequência de 7,4±8,8 meses e 9,8±14,5 meses, respectivamente, o que foi semelhante aos nossos achados, 8,9±6,4 meses. No grupo de crioablação desse estudo, 80,6% apresentavam pelo menos uma VP com lacunas de condução, com média de 2,9 lacunas por VP, que se distribuíram igualmente entre as quatro VPs. Em nosso estudo, 81,5% dos pacientes apresentavam pelo menos uma lacuna de condução de VP, com média de 6,0 lacunas por paciente e 3,6 gaps por VP. A veia com mais lacunas de condução foi a VPSE, seguida da VPSD, VPIE e VPID. A razão por trás de nossos números de lacunas mais altos pode ser explicada pela maneira como os medimos. Identificamos 12 regiões diferentes nas VPs, em oposição às oito utilizadas por outros autores. Em outros dois estudos, reconexões de VPs foram encontradas em 54 e 71% dos pacientes, e a região anterolateral da VPSE (onde a crista endocárdica pode ser encontrada) foi o local mais frequente de lacunas de condução.^14,15^ De maneira geral, a região ântero-superior das veias superiores foi o local com mais lacunas, nos estudos citados e no presente estudo. Entretanto, ao passo que a VPID foi a VP inferior com mais reconexões, nossos resultados mostraram a região ínfero-anterior como a principal fonte de reconexões, o que difere dos achados de Katering et al.,^13^ que observaram uma distribuição mais homogênea. Por fim, eletrogramas fracionados no AE foram encontrados em 29,6% dos pacientes, incidência muito superior à relatada por Galand et al.^5^

Acreditamos que nos locais em que há mais reconexões, o criobalão não tem contato adequado e, portanto, a crioterapia não atinge uma ablação mais profunda. Esse fenômeno ocorre porque o formato do ântero da VP nem sempre é circular, muitas vezes é ovalada, e as dimensões de cada veia também variam, portanto, o suporte do criobalão não é homogêneo.^14^ Além disso, uma lesão de crioablação adequada deve ter profundidade suficiente. Visto isso, locais do ântero da VP com parede mais espessa, como a junção da VPSE com o óstio do apêndice do AE (rebordo endocárdico), estão sujeitos a lesões não transmurais. Kowalski et al. confirmaram essa suposição ao mostrar em corações humanos dissecados que as lacunas de condução da VP ocorriam quando a lesão de radiofrequência não era transmural.^16^ Finalmente, uma técnica adequada é importante para obter a oclusão da veia pulmonar antes de administrar a terapia de crioablação. Diversas estratégias diferentes foram descritas. No entanto, sempre haverá casos em que o isolamento completo é impossível.^17^

Uma limitação do presente estudo reside no seu caráter descritivo. Um projeto prospectivo longitudinal forneceria mais resultados clinicamente relevantes.

## Conclusão

A incidência de lacunas na condução das VP no presente estudo foi semelhante aos achados de outros estudos. As veias pulmonares superiores apresentam a maioria das lacunas de condução e estão na região ântero-superior no apêndice da AE e na junção da VPSE, e em direção à parte do septo na VPSD. A falta de contato adequado do balão devido a variações anatômicas na VP, técnica inadequada e locais com parede espessa no ântero da VP são a base das reconexões após o procedimento inicial de ablação de FA com criobalão. Finalmente, em pacientes com FA a recorrência após IVP-CB inicial, cerca de 30% têm gatilhos, que estão no corpo do AE, principalmente na parede posterior.
