# Supplementary material for: Locais de Reconexão na Técnica de Reablação após Isolamento das Veias Pulmonares com Criobalão em Pacientes com Fibrilação Atrial Paroxística
Source: Arq Bras Cardiol. 2021 Jul 15;117(1):100–5. [Article in Portuguese] doi: 10.36660/abc.20190503 (PMC8294743; doi:10.36660/abc.20190503)
Supplement: Translation

# Translation: Reconnection Sites in Redo Ablation after Cryoballoon Pulmonary Vein Isolation in Patients with Paroxysmal Atrial Fibrillation

**Authors:** Rogelio Robledo Nolasco, Gerardo De Leon-Larios, David Eduardo Bazzini-Carranza, Elias Zavaleta, Omar Calixto-Vargas

**Affiliations:** 1 MéxicoCentro Medico Nacional 20 de Noviembre - Hemodinamia y Electrofisiologua, Ciudad de México – México

**Keywords:** Arrhythmias, Cardiac, Atrial Fibrillation, Pulmonary Veins, Radio Waves, Electrocardiography/methods, Electrocardiography, Ambulatory, Tachycardia, Ectopic Atrial, Holter

## Abstract

**Background:**

In paroxysmal atrial fibrillation (PAF), pulmonary vein isolation using cryoballoon (CB-PVI) has similar efficacy as radiofrequency ablation (RF-PVI) has. In redo ablation procedures following RF-PVI, PV reconnection is high, whereas in patients with redo following CB-PVI, information is scarce.

**Objective:**

To determine the sites of PV reconnection in patients who underwent redo ablation after initial CB-PVI.

**Methods:**

Patients who underwent an AF redo procedure, following an initial CB-PVI for PAF were included. LA electroanatomic mapping was used. A reconnection site was defined as the presence of a voltage of 0.3mV or greater in the PV and unidirectional or bidirectional conduction in the PV during sinus rhythm. Reconnections sites were identified using a clock-face view description and were ablated with radiofrequency afterwards.

**Results:**

Out of the 165 patients who underwent initial PVI, 27 required redo ablations, of which 18 (66.6%) were males, with a mean age of 55+12.3 years. The time of recurrence was 8.9+6.4 months. PV reconnection was found in 21 (77.8%) patients. There was a total of 132 conduction gaps, six per patient, 3.6 per PV. A significant number of gaps were in the anterosuperior region of the left superior PV (LSPV), and in the septal and inferior regions of the right superior PV (RSPV).

**Conclusions:**

The upper PVs had the most reconnection sites, mostly at the anterior region of the LSPV and the septal region of the RSPV. The reason behind this may be due to greater atrial wall thickness, and difficulty in achieving adequate cryoballoon contact.

## Introduction

Pulmonary vein isolation (PVI) is the cornerstone of ablative therapy in paroxysmal and persistent atrial fibrillation (AF). The most common techniques to achieve PVI are cryoballoon ablation and point-by-point radiofrequency ablation, both of which have shown similar results.^1,2^ AF recurrence following the initial PVI procedure is assumed to be mediated with pulmonary vein (PV) reconnection; since 80% of patients who undergo redo ablation show PV conduction recovery in at least one site.^3-5^ A study to determine the presence of PV reconnections in patients who underwent redo ablation after initial CB-PVI was conducted. In addition, the sites that were most likely to have PV conduction recovery were described.

## Methods

Symptomatic patients with AF who experienced recurrence after an initial cryoablation PVI, and who underwent a redo procedure, were included. Patients who received initial cryoablation therapy were those with drug-resistant AF with preserved left ventricular ejection fraction (LVEF), a left atrial anteroposterior (AP) diameter of 55 mm or less, and no evidence of left atrial (LA) appendage thrombi in a transesophageal echocardiogram.

The procedure was performed under conscious sedation, and the femoral veins were used as venous access. A decapolar catheter (Webster® Decapolar Catheter Deflectable) was positioned in the coronary sinus and an ICE-guided atrial transseptal puncture was performed using an 8F sheath (Preface Braided Guiding Sheath) and a Brockenbrough needle (BRK Transseptal Needles). A 12F steerable sheath (FlexCath®sheath Medtronic, Minneapolis, MN, USA) and a circular mapping catheter (Achieve Medtronic) were then inserted in the LA. A 28-mm cryoablation balloon catheter (Arctic Front, Cryocath^TM^, Medtronic, CA, USA or Arctic Front Advance, Minneapolis, MN) was used to deliver cryoablation therapy at the antrum of each pulmonary vein. Cryoablation therapy was delivered for 180 to 300 seconds until a minimum temperature of -40ºC was reached and PVI was ensured. Therapy was considered successful if PV entrance and exit block were both achieved. During right PV isolation, the quadripolar catheter was positioned in the superior vena cava to continuous phrenic nerve stimulation at 1,800 ms cycle length and 20 mA output to avoid hemidiaphragm paralysis.

During follow-up, an electrocardiogram and 24-hour Holter monitor (or pacemaker monitoring) were prescribed three months after the procedure and six months thereafter. Antiarrhythmic medication was suspended after the first three months if no AF or atrial tachyarrhythmias were identified. A recurrence was defined as the presence of AF or another atrial tachycardia, in an electrocardiogram strip, or during at least 30 seconds in a Holter monitor after the first follow-up visit.

Redo procedure was performed under conscious sedation, using femoral vein access, and only one atrial transseptal puncture. Anticoagulant was not suspended to perform the ablation procedure. Carto 3® System (Biosense Webster, Diamond Bar, CA, USA) and a multipolar catheter (Pentaray Nav, BiosenseWebster) were used to construct voltage maps of the LA and of each PV, using <0.3mV as a cutoff value for scar tissue and >1.0mV, for normal tissue. PV isolation was defined as absence of electrical activity inside the PV during sinus rhythm or AF, and/or the presence of entrance and exit block if the patient was in sinus rhythm.

Sites with conduction gaps were identified using a clock-face view description, as such 12 different places were considered. Once the sites of PV reconnection were located, they were ablated with radiofrequency using an irrigated contact-force sensor catheter (ThermoCool SmartTouch, Biosense Webster). In addition, fragmented electrograms were actively searched and tagged, especially in the posterior wall and roof of the LA. If the patient was in sinus rhythm, atrial pacing at a CL of 170ms was used to induce AF in order to search for fragmented electrograms, which were subsequently ablated. Ablation of the anterior wall and ridge was done at 40 W, whereas 25W was used for the posterior wall with a temperature limit of 45ºC. We used an infusion rate of 17 to 30 mL/min of normal saline, and a pressure force of 6-30g.

If the patient was in AF, ablation was considered successful if there was an impedance drop of 8-10 ohms, a decrease in amplitude, or elimination of atrial electrograms. If the patient was in sinus rhythm, ablation success was determined by loss of pacing capture at the ablation site. Lastly, ablation lines were made at the roof and mitral isthmus, and if typical atrial flutter was detected, ablation of the cavotricuspid isthmus was done as well.

### Statistical Analysis

This is a descriptive observational study. Data distribution was tested with the Shapiro Wilk normality test. Categorical variables were expressed as total number and percentages, whereas normally distributed variables were expressed as mean and standard deviation. SPSS v.20 was used for data analysis. Statistical significance was considered if p<0.05.

## Results

### Study population

From 2014 to 2018, 164 patients underwent PVI using the cryoablation balloon technique, of which 27 experienced AF recurrence and had to undergo redo procedure, after a follow up of 10.7+7.2 months. Of these patients, 18 (66.6%) were men with a mean age of 55±12.3 years, a mean CHA2DS2-VASc score of 1.9±1.6, and mean LVEF of 60.8±17.2%. Other patients’ characteristics can be seen in Table 1. Informed and written consent was required prior to the redo AF ablation procedure.

**Table 1:** – Patients’ characteristics

Men	18 (66.6)
Age (years)	55±12.3
Hypertension	15 (55.5)
Diabetes mellitus	8 (29.6)
Heart failure	1 (3.7)
History of infarction	1 (3.7)
History of stroke (%)	3 (11.1)
Pacemaker carrier(%)	3 (11.1)
Duration of AF (months)	13.2±13.5
CHA2DS-VASC	1.9±1.6
Number of antiarrhythmic drugs tested	1.2±0.6
LVEF	60.8±17.2
LA diameter (mm)	40.2±8.0
Time before recurrence (months)	8.9±6.4

*Data are expressed in numbers (%) or mean ± standard deviation. AF: atrial fibrillation; LA: Left atrium; LVEF: left ventricle ejection fraction. Data analysis was performed with SPSS v.20.*

### Redo procedure and PV reconnection

The characteristics of redo procedures are described in Table 2. The time to AF recurrence was 8.8±8.2 months. Of the 27 patients studied, AF recurrence was detected in 17 (62.9%), with Holter monitoring, seven (25.9%) with EKG, and three (11.1%) with pacemaker monitoring. Besides that, 18 (66.6%) were in sinus rhythm; the others were in AF. A reconnection site was defined as the presence of a voltage of 0.3mV or greater in the PV, and unidirectional or bidirectional conduction in the PV during sinus rhythm.

**Table 2:** – Characteristics of redo procedure

Patients with PV reconnection	21 (77.8)
Time before recurrence (months)	10.56.5
Number of veins per patient	1.6±0.4
Number of gaps per patient	6.0±0.5
Number of gaps per PV	3.6±0.3
Left superior pulmonary vein gaps	56 (42.4)
Left inferior pulmonary vein gaps	12 (9.1)
Right superior pulmonary vein gaps	35 (26.5)
Right inferior pulmonary vein gaps	29 (21.9)
Additional ablation performed	
Complex fractionated atrial electrograms	4 (14.8)
Cavotricuspid isthmus isolation	3 (11.1)
Total procedure time (minutes)	130±17
Fluoroscopy time (min)	8.5±1.7

*Data are expressed in numbers (%) or mean ± standard deviation. Data analysis was performed with SPSS v.20. VP: pulmonary vein.*

Three vein accesses were obtained in femoral veins. In all cases, only one transeptal puncture guided by intracardiac ultrasound was performed; first, the Pentaray catheter was introduced to perform a voltage and conduction map of the LA and PV. 109 PV were identified among the 27 patients who underwent redo ablation, of which 36 (33.0%) had at least one reconnection site. 22 patients (81.5%) had at least one PV with a conduction gap, with a mean of 1.6±0.4 PV per patient. Nine patients had a reconnection site in one PV (40.9%), 11 patients (50%) in two different PV, one patient (4.5%) in three PV, and one patient (4.5%) in all four PV.

### Location of PV conduction gaps

A total of 132 PV conduction gaps were observed, with a mean of 6.0±0.5 conduction gaps per patient and 3.6±0.3 gaps per PV; these reconnections were located at the following sites: 56 (42.4%) at the left superior PV, 35 (26.5%) at the right superior PV, 29 (21.9%) at the right inferior PV, and 12 (9.1%) at the left inferior PV (Figure 1). The site with the most reconnections was the junction between the LSPV and the left atrial appendage (endocardial ridge) followed by the posteroinferior region of the LSPV (71%); and, lastly, the posterior region of LSPV (29%). The LIPV had the least connection gaps, which were evenly distributed around the vein. The RSPV had the most reconnections out of the right pulmonary veins, mostly at the anterior-superior and inferior regions (94% of the total). RIPV gaps were evenly distributed around the vein, slightly favoring the inferior regions.

**Figure 1:** – Distribution of the reconnection gaps in the four PV (the number on the core of each circle). The LSPV is the most reconnected, 71% of the gaps are in the anterosuperior and anteroinferior regions. The RSPV had more reconnections in the anterosuperior and septal regions. LIPV: left inferior pulmonary vein; LSPV: left superior pulmonary vein; RIPV: right inferior pulmonary vein; RSPV: right superior pulmonary vein.

### Fragmented electrogram locations and other arrhythmias

Fragmented atrial electrograms were identified in eight (29.6%) patients, mostly in the posterior wall. Typical atrial flutter was found in nine (33.3%) patients and underwent cavotricuspid-isthmus ablation until bidirectional block was achieved.

The mean duration of the RF redo ablation procedures was 130±17 minutes, with a mean fluoroscopy time of 8.5±1.7 minutes. There were two procedure-related complications, an inguinal hematoma which underwent conservative treatment, and a pericardial effusion, which was promptly resolved following pericardial puncture.

## Discussion

Point-by-point radiofrequency pulmonary vein isolation has shown to be an effective treatment for paroxysmal AF, and as such is currently recommended in AF clinical guidelines.^6^The cornerstone of paroxysmal AF ablative therapy is PVI, which has traditionally been performed with radiofrequency.^7-9^ Most recently, cryoablation therapy emerged as a viable alternative, with similar results.^1,2^ In the Fire and Ice Study; both approaches were equally effective, especially when comparing second-generation cryoballoon with contact-force catheter.^10^

In patients who undergo a redo procedure after an initial PVI, PV connection gaps can be found in more than 95% of the time. The left pulmonary veins seem to have the most reconnections, especially at the anterior and inferior areas.^4,11,12^Katering et al.,^13^published a case series of AF redo procedures which documented an average of 2.9 PV reconnections in comparison to our findings, 1.6. Recently, another study published showed a recurrence time after PVI with cryoablation and radiofrequency of 7.4±8.8 months and 9.8±14.5 months, respectively, which was similar to our findings, 8.9±6.4 months. In the cryoablation group of such study, 80.6% had at least one PV with conduction gaps, with an average of 2.9 gaps per PV, which were equally distributed among the four PV. In our study, 81.5% of patients had at least one PV conduction gap, with a mean of 6.0 gaps per patient and 3.6 gaps per PV. The vein with most conduction gaps was the LSPV, followed by the RSPV, the LIPV, and the RIPV. The reason behind our higher gap numbers may be explained by the way we measured them. We identified 12 different regions in the PV, as opposed to the eight used by other authors. In other two studies, PV reconnections were found in 54 and 71% of patients, and anterolateral region of the LSPV (where the endocardial ridge can be found) was the most frequent site of conduction gaps.^14,15^Overall, the anterosuperior region of the superior veins was the site with the most gaps, in the aforementioned studies and in the present study. However, whereas the RIPV was the inferior PV with the most reconnections, our results showed the inferoanterior region as the main source of reconnections, which differs from the findings of Katering et al.,^13^ which saw a more homogenous distribution. Lastly, fractionated electrograms in the LA were found in 29.6% of patients, a much higher incidence than that reported by Galand et al.^5^

We believe that in places in which there are more reconnections, the cryoballoon does not have adequate contact and, therefore, cryotherapy does not reach a deeper ablation. This phenomenon occurs because PV antrum shape is not always circular, are oval in many, and the dimensions of each vein also varies, therefore the support of the cryoballoon is not homogeneous.^14^ In addition, an adequate cryoablation lesion must have sufficient depth. As such, sites of the PV antrum with a thicker wall, like junction of the LSPV and the ostium of the LA appendage (endocardial ridge) are prone to have non-transmural lesions. Kowalski et al. confirmed this assumption by showing in dissected human hearts, that PV conduction gaps occurred when the radiofrequency lesion was not transmural.^16^ Finally, an adequate technique is important to achieve pulmonary vein occlusion prior to delivering the cryoablation therapy. Several different strategies have been described. However, there will always be cases when complete isolation is impossible.^17^

One limitation of the present study relays on its descriptive nature. A longitudinal prospective design would provide further clinically relevant results.

## Conclusion

The incidence of PV conduction gaps in our study was similar to the findings of other studies. The superior pulmonary veins have the most conduction gaps and are in the anterosuperior region at the LA appendage and LSPV junction, and towards the part of the septum in the RSPV. Lack of adequate balloon contact due to anatomical variations in PV, inadequate technique and sites with thick wall in the PV antrum underlie reconnections after initial cryoballoon AF ablation procedure. Finally, in patients with AF recurrence following the initial CB-IVP, roughly 30% have triggers, which are in the body of the LA, mainly in the posterior wall.
